# Comparative Anticonvulsant Study of Epoxycarvone Stereoisomers

**DOI:** 10.3390/molecules201119649

**Published:** 2015-10-29

**Authors:** Paula Regina Rodrigues Salgado, Diogo Vilar da Fonsêca, Renan Marinho Braga, Cynthia Germoglio Farias de Melo, Luciana Nalone Andrade, Reinaldo Nóbrega de Almeida, Damião Pergentino de Sousa

**Affiliations:** 1 Instituto de Pesquisa em Fármacos e Medicamentos, Universidade Federal da Paraíba, CP 5009, João Pessoa, CEP 58051-900, PB, Brazil; paulasalgado87@gmail.com (P.R.R.S.); divilar@hotmail.com (D.V.F.); renan_braga123@hotmail.com (R.M.B.); cynthia_fariasm@hotmail.com (C.G.F.M.); reinaldoan@uol.com.br (R.N.A.); 2 Departamento de Farmácia, Universidade Federal de Sergipe, São Cristóvão-SE, CEP 49100-000, Brazil; lulisynalone@yahoo.com.br; 3 Departamento de Fisiologia e Patologia, Universidade Federal da Paraíba, CP 5009, João Pessoa, CEP 58051-900, PB, Brazil; 4 Departamento de Ciências Farmacêuticas, Universidade Federal da Paraíba, CP 5009, João Pessoa, CEP 58051-900, PB, Brazil

**Keywords:** anticonvulsant, carvone, stereoisomers, terpene, natural products, essential oils, pentylenetetrazole, seizures, *para*-menthanes, enantiomers

## Abstract

Stereoisomers of the monoterpene epoxycarvone (EC), namely (+)-*cis*-EC, (−)-*cis*-EC, (+)-*trans-*EC, and (−)-*trans*-EC, were comparatively evaluated for anticonvulsant activity in specific methodologies. In the pentylenetetrazole (PTZ)-induced anticonvulsant test, all of the stereoisomers (at 300 mg/kg) increased the latency to seizure onset, and afforded 100% protection against the death of the animals. In the maximal electroshock-induced seizures (MES) test, prevention of tonic seizures was also verified for all of the isomers tested. However, the isomeric forms (+) and (−)-*trans*-EC showed 25% and 12.5% inhibition of convulsions, respectively. In the pilocarpine-induced seizures test, all stereoisomers demonstrated an anticonvulsant profile, yet the stereoisomers (+) and (−)-*trans*-EC (at 300 mg/kg) showed a more pronounced effect. A strychnine-induced anticonvulsant test was performed, and none of the stereoisomers significantly increased the latency to onset of convulsions; the stereoisomers probably do not act in this pathway. However, the stereoisomers (+)-*cis*-EC and (+)-*trans*-EC greatly increased the latency to death of the animals, thus presenting some protection. The four EC stereoisomers show promise for anticonvulsant activity, an effect emphasized in the isomers (+)-*cis*-EC, (+)-*trans*-EC, and (−)-*trans*-EC for certain parameters of the tested methodologies. These results serve as support for further research and development of antiepileptic drugs from monoterpenes.

## 1. Introduction

Epilepsy has been characterized as a brain disease characterized by certain conditions: at least two unprovoked seizures in a range greater than 24 h; an unprovoked seizure and the likelihood of further recurrent crises after two unprovoked seizures, continuation over 10 years; and diagnosis of epileptic syndrome [1]. Epileptic patients present recurrent spontaneous seizures [2], and some 30% of them are considered resistant to drug therapy, without adequate response to treatment. Often, surgical removal of the epileptic focus is the only option that actually controls the emergence of seizures [3]. Degradation of cognitive functions (due to factors such as seizure etiology, and type), psychosocial problems [4], and possible psychomotor impairment [5] are clinically observed in these patients.

It is known that the existing clinical anti-epileptic drugs are not good anti-epileptogenics, since they only increase the onset threshold for seizure [6]. Many patients have seizures despite access to the complete pharmacological arsenal [7]. When considering the development of new antiepileptic drugs from 1981–2002, it is important to emphasize the role of natural products, since most of the synthetic drugs developed have had a natural product as their model. This reinforces the role of nature as a source for anticonvulsant agents [8].

Essential oils and their constituents are natural products known for their biological effects [9], including anxiolytic [10], spasmolytic [11], antinociceptive [12], and anticonvulsant [13] activities. Structural diversity in the constituents of essential oils is responsible for the variety of biological effects already observed; this is especially true for the monoterpene class, which has shown interesting anticonvulsant activity in animal models [14]. Besides their functional groups, monoterpenes have a range of optical isomers of specific compounds; these isomers may have different properties involving differing pathways [15], with synergistic and/or antagonistic actions [16], and membrane models [17].

Epoxycarvone is a monoterpene found in essential oils of plants such as *Carum carvi* [18], *Kaempferia galanga* [19], and others [20], or it is obtained by organic synthesis [21]. Epoxycarvone has demonstrable antimicrobial activity against *Staphylococcus aureus* and *Candida albicans* [22], and pharmacological effects on the central nervous system [23], with antinociceptive [24], and anticonvulsant activities [25]. The compound has four isomers, whose psychopharmacological stereo-selectivity is still unknown. These stereoisomers can be obtained using the enantiomers of carvone as starting materials; they also have differences in anticonvulsant activity due to the influence of their stereogenic center configuration [26]. This study aims to comparatively verify the anticonvulsant potential of the four stereoisomers of epoxycarvone in animal models of chemically and electrically induced seizures.

## 2. Results and Discussion

### 2.1. Preparation of Epoxycarvone Stereoisomers

**Scheme 1:**
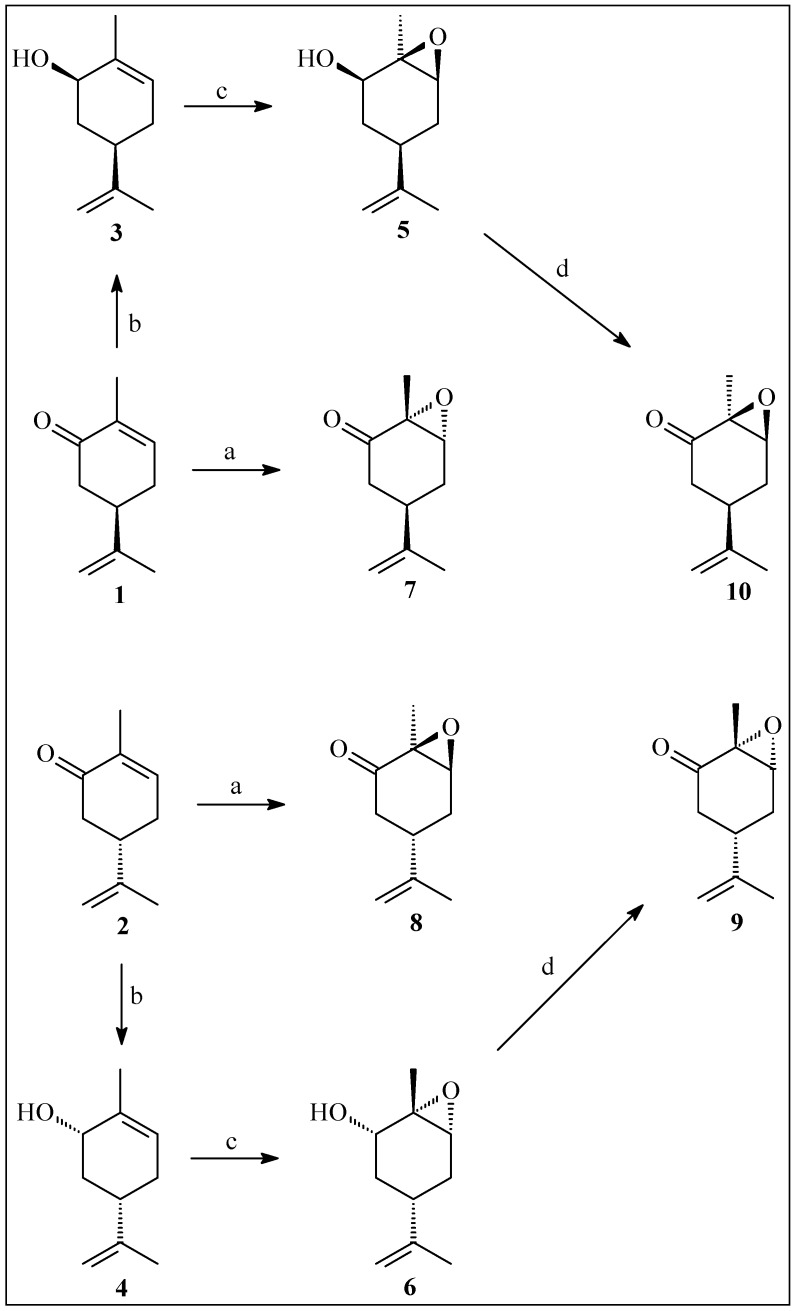
Preparation of epoxycarvone stereoisomers. *Reagents and Conditions*: a. H_2_O_2_ 30%, KOH, MeOH, 4 h, 0 °C; b. NaBH_4_, CeCl_3_**^.^**7 H_2_O, MeOH, 5 min., 20 °C; c. *m*-CPBA in CH_2_Cl_2_, 6 h, 0 °C; d. PCC, Pyr, 30 h, r.t.

The enantiomer *trans*-carvone oxides **9** and **10** were prepared via stereoselective reduction of the carbonyl group of carvones **1** and **2**, stereospecific hydroxyl-assisted epoxidation of the allylic alcohols **3** and **4** and oxidative regeneration of the carbonyl group from epoxy alcohols **5** and **6** (Scheme 1).

Alkaline epoxidation of carvones **1** and **2** formed enantiomer *cis*-carvone oxides **7** and **8**. The ^13^C- and ^1^H-NMR, IR, polarimetric analysis, and comparison of the data with that found in the literature [27,28,29,30] confirmed the chemical structures and stereochemistry of the carvone oxides and intermediates.

### 2.2. Pentylenetetrazole-Induced Seizure Test

In the acute test of pentylenetetrazole-induced seizures, (+)-*cis*-EC (**7**): 900.0 ± 0.0 s, (−)-*cis*-EC (**8**): 763.3 ± 69.2 s, (+)-*trans*-EC (**9**): 791.1 ± 108.9 s and (−)-*trans-*EC (**10**): 743.0 ± 83.7 s, significantly increased the latency to onset of first seizure, compared with the control group (109.9 ± 13.0 s) (Table 1). Similar response to the groups treated with the isomers was seen in animals treated with diazepam (4 mg/kg) (900.0 ± 0.0 s). Table 1 presents data related to the first seizure latency to onset, and to the score for dead animals after treatments. No deaths were recorded in the group treated with epoxycarvone stereoisomers (300 mg/kg) and the standard antiepileptic drug, diazepam (4 mg/kg), in contrast to what was observed for the control group.

**Table 1:** Epoxycarvone stereoisomers effect on pentylenetetrazole-induced seizures test.

Experimental Groups	Latency (s)	Mortality (%)
Control (Tween 80 5%)	109.9 ± 13.0	50
Diazepam (standard)	900.0 ± 0.0 ^a^	0
(+)-*cis*-EC (**7**)	900.0 ± 0.0 ^a^	0
(−)-*cis*-EC (**8**)	763.3 ± 69.3 ^a^	0
(+)-*trans*-EC (**9**)	791.1 ± 108.9 ^a^	0
(−)-*trans*-EC (**10**)	743.0 ± 83.7 ^a^	0

The values represent the mean ± SEM (*n* = 8 in each group). ^a^ *p* < 0.001 for the epoxycarvone stereoisomers group (300 mg/kg) as compared to the control group (ANOVA One way/Tukey’s test). Fisher’s exact test was used to analyze the mortality rate.

### 2.3. Maximal Electroshock-Induced Seizure (MES)

The seizure durations were decreased (Table 2) in the groups treated with (**7**: 9.8 ± 0.5 s), (**8**: 8.3 ± 1.4 s), (**9**: 0.7 ± 0.7 s) and (**10**: 0.0 ± 0.0 s) compared to the control group (16.3 ± 0.9 s). As expected, animals that received the standard drug phenytoin (25 mg/kg) also presented seizures inhibition of (0.0 ± 0.0 s).

**Table 2:** Epoxycarvone stereoisomers effect on maximal electroshock-induced seizures test (MES).

Experimental Groups	Seizures Duration (s)	Tonic Seizures (%)	Mortality (%)
Control (Tween 80 5%)	16.3 ± 0.9	100	37.5
Phenytoin (standard)	0.0 ± 0.0 ^a^	0	0
(+)-*cis*-EC (**7**)	9.8 ± 0.5 ^a^	100	0
(−)-*cis*-EC (**8**)	8.3 ± 1.4 ^a^	87.5	0
(+)-*trans*-EC (**9**)	0.7 ± 0.7 ^a^	25	0
(−)-*trans*-EC (**10**)	0.0 ± 0.0 ^a^	12.5	0

The values represent the mean ± SEM (*n* = 8 in each group). ^a^ *p* < 0.001 for the epoxycarvone stereoisomers group (300 mg/kg) as compared to the control group (ANOVA One way/Tukey’s test). Fisher’s exact test was used to analyze the percentages of animals with seizures and the mortality rate.

In Table 2, the tonic convulsions percentage was significant in groups treated with (**7**: 100%) and (**8**: 87.5%). The groups which received (**9**: 25%), and (**10**: 12.5%) presented fewer tonic convulsions. The control (100%) and standard (0%) groups had the expected responses. No group treated with either the isomers or phenytoin showed mortality, the control group showed 37.5%.

### 2.4. Pilocarpine-Induced Seizures Test

The results for the behavioral changes induced by administration of pilocarpine (400 mg/kg) in animals treated with isomers (300 mg/kg), vehicle, and diazepam (4 mg/kg) are shown in Table 3.

**Table 3:** Epoxycarvone stereoisomers effect on pilocarpine-induced seizures test.

Experimental Group	Latency to Convulsions (s)	Latency to Death (s)	Peripheral Cholinergic Signs (%)	Stereotyped Movements (%)	Tremors (%)	Seizures (%)	*Status Epilepticus* (%)	Mortality (%)
Control (Tween 80 5%)	429.3 ± 25.4	817.6 ± 22.3	100	100	100	100	100	100
Diazepam (standard)	3600.0 ± 0.0 ^b^	3600.0 ± 0.0 ^b^	100	0	50	12.5	0	0
(+)-*cis*-EC (**7**)	862.6 ± 36.6 ^b^	1845.9 ± 453.1	100	50	100	100	0	75
(−)-*cis*-EC (**8**)	890.8 ± 76.2 ^b^	1135.9 ± 68.1	100	100	100	100	50	100
(+)-*trans*-EC (**9**)	1044.0 ± 49.1 ^b^	2895.7 ± 343.3 ^b^	50	100	50	100	50	50
(−)-*trans*-EC (**10**)	888.4 ± 78.3 ^b^	2345.0 ± 477.9 ^a^	50	100	50	100	50	50

The values represent the mean ± SEM (*n* = 8 in each group). ^a^ *p* < 0.05; ^b^ *p* < 0.001 for the epoxycarvone stereoisomers group (300 mg/kg) as compared to the control group (ANOVA One way/Tukey’s test). Fisher’s exact test was used to analyze the percentages of animals with peripheral cholinergic signs, stereotyped movements, tremors, seizures, *status epilepticus* and the mortality rate.

All groups showed peripheral cholinergic signs, but the animals treated with (**9**: 50%) and (**10**: 50%), both 300 mg/kg, showed fewer percentages of animals with these signs. The control and experimental groups had convulsions (100%), while the standard group showed a lower percentage for this parameter (12.5%). The death percentage was highest in animals treated with vehicle (100%) and (**8**: 100%). The animals that received diazepam (4 mg/kg) did not die during the experiment. All of the animals had tremors, but in animals treated with (**9**: 50%) and (**10**: 50%), the parameter was smaller. Regarding emergence of *status epilepticus*, the group treated with (**7**: 0%) did not score, while 100% of the control groups did. Pilocarpine (400 mg/kg) induced stereotypical movements in all groups, except the standard group, which received diazepam (0%). Latency to the first seizure (Table 3) increased significantly in animals that received (**7**: 862.6 ± 36.6 s), (**8**: 890.8 ± 76.2 s), (**9**: 1044.0 ± 49.1 s), (**10**: 888.4 ± 78.3 s) compared to the control group (429.3 ± 25.4 s). A similar result was observed for latency to death in the animals (Table 3), where those treated with (**9**: 2895.7 ± 343.3 s) and (**10**: 2345.0 ± 478.0 s), both at 300 mg/kg, increased latency compared to the control group (817.6 ± 22.3 s). There was a significant difference between the groups treated with (**8**: 1135.9 ± 68.1 s) and (**9**: 2895.7 ± 343.3 s), and no significant increase for this parameter in animals from groups **7** and **8** (300 mg/kg).

### 2.5. Strychnine-Induced Seizure Test

No significant differences were observed in latency to the first seizure in animals treated with the EC isomers (300 mg/kg) or the vehicle, in the strychnine-induced seizures model. However, there was a significant increase in latency to death of the animals treated (**7**: 306.5 ± 55.3 s) and (**9**: 396.5 ± 61.5 s), both 300 mg/kg, as compared to the control group (53.0 ± 0.6 s). All animals had seizures after strychnine administration, and there were no survivors except for the group treated with diazepam (4 mg/kg) (Table 4).

**Table 4:** EC isomers effect on strychnine-induced seizures test.

Experimental Group	Latency to Convulsions (s)	Latency to Death (s)	Seizures (%)	Mortality (%)
Control (Tween 80 5%)	51.1 ± 0.5	53.0 ± 0.6	100	100
Diazepam (standard)	144.3 ± 29.4 ^a^	435.8 ± 93.2 ^b^	100	0
(+)-*cis*-EC (**7**)	79.2 ± 20.8	306.5 ± 55.3	100	100
(−)-*cis*-EC (**8**)	89.2 ± 9.1	169.3 ± 17.8	100	100
(+)-*trans*-EC (**9**)	111.8 ± 21.8	396.5 ± 61.5 ^c^	100	100
(−)-*trans*-EC (**10**)	65.0 ± 4.9	273.8 ± 51.4	100	100

The values represent the mean ± SEM (*n* = 8 in each group). ^a^ *p* < 0.001; ^b^ *p* < 0.01; ^c^ *p* < 0.05 regarding the epoxycarvone stereoisomers group as compared to the control group (ANOVA One way/Tukey’s test). Fisher's exact test was used to analyze the percentages of animals with seizures, and the mortality rate.

In this study, the anticonvulsant activity of the stereoisomers (+)-*cis*-EC (**7**), (−)-*cis*-EC (**8**), (+)-*trans*-EC (**9**), and (−)-*trans*-EC (**10**) were analyzed comparatively in four methodologies that induced seizures chemically and electrically. Pentylenetetrazole-induced seizure test is one of the standard methods of anticonvulsant screening [31]. In this experiment, the isomers **7**, **8**, **9**, and **10** (300 mg/kg) increased the latency to onset of seizure. This finding emphasizes the anticonvulsant potential attributed to the 4 isomers. The protection also extended to the animal mortality rate; there were no deaths in groups treated with the isomers, or in the standard group (diazepam). Anticonvulsants such as diazepam and phenobarbital have the ability to open GABA_A_ channels, inhibiting seizures induced by PTZ [32], a substance that acts as an antagonist of the GABA_A_ receptor [33]. The activation of GABAergic neurotransmission inhibits or attenuates seizures, while inhibition of this neurotransmission increases convulsions [34]. Idris and collaborators [35] evaluated the anticonvulsant profile of three isomers (30 mg/kg) of *N*-benzyl-3-[(methylphenyl)amino]propanamide, and all demonstrated excellent anti-convulsant properties in the pentylenetetrazole-induced seizure test. Another study by Wieland [36] compared the effect of two neuro-active endogenous steroid isomers, 3α-hydroxy-5α-pregnan-20-one and 3α-hydroxy-5β-pregnan-20-one, for possible behavioral differences in this methodology. The resulting anticonvulsant activity was similar, which reinforces the findings of the present study. Seizure inhibition in the PTZ test by all four isomers, suggests possible interference in GABAergic neuro-transmission.

Maximal electroshock-induced seizures (MES) testing was also performed. This model is widely used to study anticonvulsant agents [37]. In the MES test, drugs that suppress tonic-clonic seizures have the ability to prevent the spread of seizure discharge through neuronal tissue, and thus increase the seizure threshold [38]. There were differences in the EC isomers effects, although all of the isomers significantly reduced the duration of the seizures compared to the control group. Substances **9** and **10** showed greater reductions. In other studies, constitutional isomers of valproic acid amide derivatives were comparatively tested for anticonvulsant activity in the MES test, with negligible modulations occurring [39]. *trans*-1-Propenyl-2,4,5-trimethoxy-benzene, also known as α-asarone, showed anticonvulsant property in the above test [40]. As previously reported, the monoterpene α,β-epoxycarvone prevented tonic seizures induced in this test [25], as was seen with the four isomers. Certain enantiomers of neurosteroids [41] also show remarkable anticonvulsant effects in the MES test, supporting the results of this research.

In both the PTZ and MES test all of the isomers showed significant protection (increased time to onset of convulsions). These data emphasize their anticonvulsant potential, and suggests that further studies regarding the possible participation of pathways involving the GABA_A_ receptor, must be performed. A study by Almeida and collaborators [25] showed that the anticonvulsant effect of monoterpene α,β-epoxycarvone was not reversed by pretreatment with flumazenil, a selective GABA_A_ receptor antagonist. Since α,β-epoxycarvone was the isomer analyzed in the comparative study and knowing this information about its mechanism of action, it was suggested that the possible role in GABAergic receptors might occur in other subunits involved with epileptogenesis in both animal models [42] and in humans [43].

Epilepsy pathophysiology has been understood using animal models, which induce seizures. One model that reproduces human epilepsy components is the pilocarpine-induced seizures test [44]. Pilocarpine is a non-selective agonist of muscarinic acetylcholine receptors; and brain structures with a high density of muscarinic receptors play an important role in the emergence of seizures induced by this chemical agent [45]. Following the seizures induced by pilocarpine administration there is a latency period preceding spontaneous and recurrent focal seizures. Neuropathological changes, such as hippocampal sclerosis occur with *status epilepticus* (induced by pilocarpine), similar to the temporal lobe epilepsy in humans [46]. The animals treated with the EC isomers (300 mg/kg) showed signs of peripheral cholinergic activity; however, greater protection was afforded by isomers **9** and **10**, reducing data percentage of cholinergic signs. Regarding the emergence of *status epilepticus*, **7** was the most protective. The decrease in the tremors (as seen with **9** and **10**, the increase in time to onset of seizures (as seen with all isomers), and protection against death (observed in the groups treated with **7**, **9** and **10** are of great interest for developing new antiepileptic drugs. Significant differences in latency to death were observed (for the first time) between isomers, where **9** was more protective than **8**. The results suggest in this methodology, that isomer **9** was found to be the most promising, since it was significantly effective in increasing the latency to onset of seizures. Protection in latency to death of the animals was also observed. Substance **7**, as well as **8**, showed significant results only in increased latency to onset of convulsions, and not for latency to death. The animals treated with **7** and **8**, though increasing time to the first seizures, showed no protective effects for latency to death. As a comparative study of test substances, the results presented stereoisomer **9** as the most promising. These initial findings with this methodology are not enough to suggest muscarinic antagonism as the source of their anticonvulsant action, but do reinforce the potential of stereoisomers **7**, and especially **9**, and **10** for future research for the treatment of epilepsy. It was seen that all of the isomers are effective in relation to anticonvulsant activity, showing only slight differences between them in certain parameters. Stereochemical differences between the isomers did not significantly affect their pharmacodynamics in the tested methodologies. A constitutional isomer of valprimide has been shown effective in the pilocarpine-induced seizures test [47]. Another study of chiral derivatives of pyrrolo[1,2-α] pyrazine (in the pilocarpine-induced seizures test) noted one derivative as the more active among the series in preventing *status epilepticus* [48], differing from the previous findings where all tested substances with structural similarity, also had similar pharmacological effects.

In the strychnine-induced seizures test, protection in animals treated with isomers regarding the latency to onset of convulsions was not observed, presenting the possibility that the compound does not act on spinal cord glycine receptors [49]. Strychnine, an important post-synaptic inhibitory transmitter, acts as a convulsing agent by competitive antagonism with glycine receptors, [50]. In contrast, it was found that the isomers **7** and **9** (at 300 mg/kg), although possibly not affecting glycine receptors, showed an increase in latency to death by still unknown mechanisms. The structural isomers and enantiomers of pinene, a monoterpene with phytotoxic action, manifested differences in physiological parameters as related to germination and corn growth, probably due to differing mechanisms of action [51]. Studies like this, involving isomeric comparisons, are important since the characteristics of the substances are evaluated, allowing the future, the development of new drugs.

Electrophysiological experiments don’t conducted to corroborate the effectiveness of EC isomers, although in a study conducted by Almeida and collaborators [25], only (+)-*cis*-EC was tested, which reduced neural excitability. The use of other isomers for this purpose may confirm (or not) involvement of the same in voltage-gated Na^+^ channels blockade. What we can safely say about these stereoisomers is that behavioral seizures are affected, as seen in the pharmacological experiments. These data are preliminary and require further study of the mechanism of action to indicate how such neuroprotection occurs.

Despite the fact the dose tested in these methodologies (300 mg/kg) is relatively high, previous studies conducted by Sousa [23] showed a high LD_50_ for one of the isomers of the epoxycarvone used in this work. Due to structural similarity between four substances, this data suggests a possible low toxicity and safety at the chosen dose for all stereoisomers. Research shows that the use of high doses are not necessarily related to high toxicity, as other studies that also have reports of substances with low toxicity, high doses with pharmacological activity and similarity to the isomers [52,53,54,55,56,57].

## 3. Experimental Section

### 3.1. Reagents

The epoxycarvone isomers were obtained as described [21,27,28,29,30], and dissolved in a Tween 80 5% emulsion. Pentylenetetrazole, pilocarpine, strychnine, and Tween 80 were purchased from Sigma (St. Louis, MO, USA).

### 3.2. Preparation of Epoxycarvone Stereoisomers

The oily compounds were prepared in our laboratory according to the literature [27,28,29,30]. The ^1^H- and ^13^C-NMR measurements were obtained with a Mercury-Varian spectrometer (Palo Alto, CA, USA) operating at 200 MHz (for ^1^H), and 50 MHz (for ^13^C). The infrared spectra were recorded on a Bomen Michelson model 102 FTIR (Bomen, Chicago, IL, USA) and the most intense or representative bands reported (in cm^−1^). Optical rotations were measured on an Optical Activity AA-10 automatic polarimeter (Optical Activity Limited, Ramsey, UK) at ambient temperature.

#### 3.2.1. Reduction of (−)-Carvone (**1**) and (+)-Carvone (**2**)

Sodium borohydride (2.5 g, 66.1 mmol) was added at 20 °C to a solution of **1** or **2** (10 g, 67 mmol), and CeCl_3_·7H_2_O (25 g, 148.5 mmol) in methanol (500 mL). The mixture was stirred for 5 min. Then, diethyl ether (100 mL) and water (100 mL) were added. The organic layer was separated, and the aqueous layer was extracted with diethyl ether (3 × 100 mL). The organic layers were combined and dried over Na_2_SO_4_, and filtered. The filtrate was concentrated under reduced pressure, and the residue was subjected to column chromatography on silica gel using a mixture of ethyl hexane and ethyl acetate (8:2) as eleuent. (−)-*cis*-Carveol (**3**) and (+)-*cis*-carveol (**4**) were obtained with 80% (53.46 mmol) and 78% (52.0 mmol) yields, respectively [27,28].

(**3**): ${\left[\alpha \right]}_{D}^{29}$ = −33.7 (CHCl_3_, *c* 0.03); IR (KBr) ν_max_: 3461, 2945, 2900, 1650, 1500, 1050, 900 cm^–1^; ^1^H-NMR (CDCl_3_): δ 5.44–5.39 (1H, m), 4.93 (2H, s), 4.25–4.21 (1H, dd, *J* = 8 Hz), 2.42–2.26 (5H, m), 2.22–2.12 (3H, m), 1.65 (3H, s), 1.56 (1H, s); ^13^C-NMR (CDCl_3_) δ: 146.3, 134.2, 125.3, 106.5, 68.7, 38.3, 36.7, 28.2, 27.2, 26.5. CAS 7632-16-8.

(**4**): ${\left[\alpha \right]}_{D}^{29}$ = +31.9 (CHCl_3_, *c* 0.03); IR (KBr) ν_max_: 3460, 2945, 2900, 1640, 1500, 1050, 880 cm^–1^; ^1^H-NMR (CDCl_3_): δ 5.45–5.33 (1H, m), 4.87 (2H, s), 4.22–4.19 (1H, dd, *J* = 8 Hz), 2.46–2.35 (5H, m), 2.17–2.08 (3H, m), 1.62 (3H, s), 1.58 (1H, s); ^13^C-NMR (CDCl_3_) δ: 147.8, 136.3, 123.1, 104.7, 69.5, 39.6, 36.5, 28.5, 27.2, 26.4. CAS 1197-06-4.

#### 3.2.2. Epoxidation of (−)-*cis*-Carveol (**3**) and (+)-*cis*-Carveol (**4**)

A solution of **3** or **4** (6 g, 39.5 mmol) in CH_2_Cl_2_ (20 mL) was added to a solution of *m*-chloroperbenzoic acid (9.51 g, 80%, 55.11 mmol) in CH_2_Cl_2_ (100 mL), and the resultant mixture was left at rest for 6 h. The filtered solution was washed with 10% aqueous NaHSO_3_, (2 × 10 mL), with 10% aqueous NaHCO_3_, (2 × 10 mL) and concentrated on a rotary evaporator. The product was purified by column chromatography on silica gel, using a mixture of hexane and ethyl acetate (9:1) as eluent. (−)-Carveol epoxide (**5**) and (+)-carveol epoxide (**6**) were obtained with 67% (26.42 mmol), and 65% (25.6 mmol) yields, respectively [28,29].

(**5**): ${\left[\alpha \right]}_{D}^{29}$ = −17.6 (CHCl_3_, *c* 0.05); IR (KBr) ν_max_: 3485, 2990, 2350, 1600, 1375, 900 cm^–1^; ^1^H-NMR (CDCl_3_): δ 5.44–5.39 (1H, m), 4.93 (2H, s), 4.25–4.21 (1H, dd, *J* = 8 Hz), 2.42–2.26 (5H, m), 2.11–1.98 (3H, m), 1.65 (3H, s), 1.56 (1H, s); ^13^C-NMR (CDCl_3_): δ 146.3, 134.2, 125.3, 106.5, 68.7, 38.3, 36.7, 28.2, 27.2, 26.5. CAS 24120-79-4.

(**6**): ${\left[\alpha \right]}_{D}^{29}$ = +16.8 (CHCl_3_, *c* 0.05); IR (KBr) ν_max_: 3500, 2980, 2350, 1600, 1350, 900 cm^–1^; ^1^H-NMR (CDCl_3_): δ 5.48–5.31 (1H, m), 4.87 (2H, s), 4.33–4.14 (1H, dd, *J* = 8 Hz), 2.53–2.35 (5H, m), 2.17–1.90 (3H, m), 2.15–2.01 (3H, m), 1.62 (1H, s), 1.58 (s); ^13^C-NMR (CDCl_3_) δ: 147.8, 136.3, 123.1, 104.7, 69.5, 39.6, 36.5, 28.5, 27.2, 26.4. CAS 39903-74-7.

#### 3.2.3. Oxidation of (−)-Carveol Epoxide (**5**) and (+)-Carveol Epoxide (**6**)

A CrO_3_-pyridine complex (7.5 g, 42 mmol) was added to a solution of compound **5** or **6** (2 g, 11.90 mmol) in pyridine (125 mL), and the resultant mixture was stirred for 30 h. The solvent was then concentrated on a rotary evaporator. The product was purified by column chromatography on silica gel, a mixture of hexane and ethyl acetate (9:1) being used as eluent. (−)-*trans*-Carvone oxide (**9**) and (+)-*trans*-carvone oxide (**10**) were obtained in 40% (5.35 mmol) and 45% (4.76 mmol) yield, respectively [28].

(**9**): ${\left[\alpha \right]}_{D}^{29}$ = −33.2 (CHCl_3_, *c* 0.01); IR (KBr) ν_max_: 3020, 2985, 1700, 880 cm^–1^; ^1^H-NMR (CDCl_3_z): δ 4.69–4.65 (2H, m), 3.38 (1H, d, *J* = 4.5), 2.74 (1H, dd, *J* = 15.6, *J* = 10.2), 2.48–2.60 (1H, m), 2.04–2.17 (1H, m), 2.02–2.16 (1H, m), 1.95 (1H, dd, *J* = 11.6, *J* = 15.2), 1.63 (3H, s), 1.33 (3H, s); ^13^C-NMR (CDCl_3_) δ: 206.5, 146.0, 110.3, 64.3, 58.3, 42.6, 41.4, 27.9, 18.8, 15.4. CAS 39903-98-5.

(**10**): ${\left[\alpha \right]}_{D}^{29}$ = +32.1 (CHCl_3_, *c* 0.01); IR (KBr) ν_max_: 3025, 2975, 1720, 880 cm^–1^; ^1^H-NMR (CDCl_3_): δ 4.70–4.64 (2H, m), 3.36 (1H, d, *J* = 4.5), 2.75 (1H, dd, *J* = 15.8, *J* = 10.1), 2.49–2.61 (1H, m), 2.03–2.15 (1H, m), 2.02–2.14 (1H, m), 1.96 (1H, dd, *J* = 11.3, *J* = 15.4), 1.63 (3H, s), 1.35 (3H, s); ^13^C-NMR (CDCl_3_) δ: 206.7, 145.0, 110.6, 62.0, 59.4, 42.4, 41.5, 28.4, 20.2, 15.7. CAS 18383-49-8.

#### 3.2.4. Epoxidation of (−)-Carvone (**1**) and (+)-Carvone (**2**)

A solution of **1** or **2** g (5 g; 33.33 mmol) was dissolved in MeOH (35 mL), 30% H_2_O_2_ (1021 mL; 9999 mmol) and 6N KOH (16.64 mL; 99.99 mmol), and the mixture was stirred for 4 h at 0 °C. The two-phase system was vigorously stirred at room temperature, and the phases were separated; the aqueous layer was extracted with Et_2_O (3 × 20 mL). The organic phase was washed with water (3 × 20 mL), and dried with Na_2_SO_3_. The solvent was evaporated and the residue chromatographed on silica gel, using a mixture of hexane and ethyl acetate (9:1) as eluent. (+)-*cis*-epoxycarvone (**7**) and (−)-*cis*-epoxycarvone (**8**) were obtained in 76% (25.33 mmol) and 69% (23.0 mmol) yield, respectively [29,30].

(**7**): ${\left[\alpha \right]}_{D}^{29}$ = +30.5 (CHCl_3_, *c* 0.03); IR (KBr) ν_max_: 2990, 2900, 1720, 1675, 880 cm^–1^; ^1^H-NMR (CDCl_3_): δ 4.69 (2H, m), 3.38 (1H, d, *J* = 4.5), 2.48–2.60 (1H, m), 2.04–2.17 (1H, m), 1.95 (1H, dd, *J* = 11.6), 1.70 (3H, s), 1.63 (3H, s); ^13^C-NMR (CDCl_3_) δ: 206.2, 146.4, 111.2, 63.2, 58.5, 41.8, 35.2, 28.8, 20.9, 15.7. CAS 36616-60-1.

(**8**): ${\left[\alpha \right]}_{D}^{26}$ = −33.4 (CHCl_3_, *c* 0.03); IR (KBr) ν_max_: 2986, 2911, 1720, 1675, 890 cm^–1^; ^1^H-NMR (CDCl_3_): δ 4.71 (2H, m), 3.39 (1H, d, *J* = 4.5), 2.49–2.59 (1H, m), 2.03–2.17 (1H, m), 1.97 (1H, dd, *J* = 11.5), 1.70 (3H, s), 1.63 (3H, s); ^13^C-NMR (CDCl_3_) δ: 205.7, 146.8, 111.6, 63.2, 57.4, 41.4, 36.5, 28.8, 20.9, 15.5. CAS 39903-97-4.

### 3.3. Experimental Section

#### 3.3.1. Animals

Swiss albino male mice (*Mus musculus*), 2–3 months old, weighing from 25 to 35 g were used. The animals were housed in polyethylene cages and maintained under controlled temperature (21 ± 1 °C), with a 12 h light/dark cycle (lights on at 6:00 a.m.), with food (Purina^®^ commercial pellet feed, Paulínia, São Paulo, Brazil), and water available *ad libitum* until 1 h prior to tests. The tests were performed in the period between 12:00 and 17:00; mice were placed in polyethylene cages with 4 animals each and moved to the place of testing at least 1 h before tests to minimize eventual behavioral alterations. Each animal was used only once and euthanized at the end of the test. All experimental procedures were analyzed and previously approved by the Ethics Committee on Animal Use (CEUA) UFPB, under Certificate No. 0109/11.

#### 3.3.2. Behavioral Experiments

##### Pentylenetetrazole-induced Seizure Test

Briefly, the test animals were divided into six groups (*n* = 8): The experimental groups were treated with (+)-*cis*-epoxycarvone (**7**), (−)-*cis*-epoxycarvone (**8**), (+)-*trans*-carvone oxide (**9**), (−)-*trans*-carvone oxide (**10**) (300 mg/kg, i.p.), the control group received Tween 80 5% (10 mL/kg) and the standard group was treated with diazepam (4 mg/kg, i.p.). After 30 min of treatment, the mice were injected with pentylenetetrazole (75 mg/kg, i.p.), and each animal was observed for onset of first convulsion (first generalized clonic seizure with loss of righting reflex) and mortality over 15 min [31,58].

##### Maximal Electroshock-induced Seizure (MES)

Similarly to the previous tests, in a single trial, animals were divided into six groups (*n* = 8): The experimental groups were treated with (+)-*cis*-epoxycarvone (**7**), (−)-*cis*-epoxycarvone (**8**), (+)-*trans*-carvone oxide (**9**), (−)-*trans*-carvone oxide (**10**) (300 mg/kg, i.p.), the control group received a Tween 80 5% emulsion and the standard group was treated with phenytoin (25 mg/kg, i.p.). After 30 min, the seizures were induced by a pair of auricular clip electrodes by means of an electroshock of 150 pulses/s and 0.5 s duration (ECT UNIT 7801). The main parameters evaluated were: the number of animals with tonic seizures (complete hind limb extensions), death scores, and seizure duration [32,59].

##### Pilocarpine-induced Seizure Test

In this test, animals were divided into six groups (*n* = 8): The experimental groups were treated with (+)-*cis*-epoxycarvone (**7**), (−)-*cis*-epoxycarvone (**8**), (+)-*trans*-carvone oxide (**9**), (−)-*trans*-carvone oxide (**10**) (300 mg/kg, i.p.), the control group received Tween 80 5% (10 mL/kg), and the standard group was treated with diazepam (4 mg/kg, i.p.). After 30 min of treatments, the animals received pilocarpine (400 mg/kg, i.p.). To compare seizure severity after pilocarpine administration, in a period of 1 h, mice were observed with appearance of peripheral cholinergic signs (miosis, diarrhea, and urination), stereotyped movements (continuous sniffing, paw licking, and rearing), tremors, seizures, status epilepticus, mortality, latency to onset seizures and death [33,34,60,61].

##### Strychnine-induced Seizure Test

The animals were divided into six groups (*n* = 8): The experimental groups were treated with (+)-*cis*-epoxycarvone (**7**), (−)-*cis*-epoxycarvone (**8**), (+)-*trans*-carvone oxide (**9**), (−)-*trans*-carvone oxide (**10**) (300 mg/kg, i.p.), the control group received Tween 80 5% (10 mL/kg) and the standard group was treated with diazepam (4 mg/kg, i.p.). After 30 min of the above treatment, animals of all groups were treated with strychnine (20 mg/kg, i.p.), the latency for the onset of first seizure and death as well as the seizure and mortality rate were evaluated [35,36].

#### 3.3.3. Statistical Analysis

The results presented were obtained using ANOVA, followed by Tukey’s *post-hoc* test. Data were analyzed using the GraphPad Prism program version 5.00 (GraphPad Sotware Incorporated, San Diego, CA, USA), and the values obtained, were expressed as the mean ± standard error of mean. Fisher’s exact test was used to analyze the percentages of animals with peripheral cholinergic signs, stereotyped movements, tremors, seizures, *status epilepticus* and the mortality rate. Differences were considered statistically significant when *p* < 0.05.

## 4. Conclusions

The results indicate that the four epoxycarvone stereoisomers tested are active in several seizure models, effectively decreasing tonic seizures, and also in increasing the animal survival rate after induced seizures. The differences in isomer stereochemistry exerted little influence on pharmacological activity in some of the animal models used. Further studies should be conducted to characterize the mechanisms of action of these four epoxycarvone isomers, and to clarify the structure-activity relationships of the tested compounds.
